# Comparison of Expectant and Excisional/Ablative Management of Cervical Intraepithelial Neoplasia Grade 2 (CIN2) in the Era of HPV Testing

**DOI:** 10.1155/2022/7955290

**Published:** 2022-03-24

**Authors:** Kevin Dominique Tjandraprawira, Adeola Olaitan, Aviva Petrie, Nafisa Wilkinson, Adam N. Rosenthal

**Affiliations:** ^1^Elizabeth Garrett Anderson's Institute for Women's Health, University College London, London, UK; ^2^Department of Obstetrics and Gynaecology, Faculty of Medicine, Universitas Padjadjaran, Bandung, Indonesia; ^3^University College London Hospitals NHS Trust, London, UK; ^4^Eastman Dental Institute, University College London, UK

## Abstract

**Objective:**

To investigate conservative and excisional/ablative treatment outcomes for cervical intraepithelial neoplasia grade 2 (CIN2) following introduction of virological test of cure.

**Methods:**

This was a retrospective study of prospectively collected data at a teaching hospital colposcopy unit. 331 sequential biopsy-proved CIN2 cases were involved. CIN2 cases diagnosed between 01/07/2014 and 31/12/2017 were either conservatively managed or treated with excision/ablation and then were followed up until discharge from colposcopy clinic and then using the national cervical cytology database. Outcomes were defined: cytological/histological regression was absence of high-grade CIN on biopsy and/or high-grade dysplasia; virological regression was cytological/histological regression and negative human papillomavirus testing; persistence was biopsy-proven CIN2 and/or moderate dyskaryosis; progression was biopsy-proven CIN3+ and/or severe dyskaryosis.

**Results:**

Median follow-up was 22.6 months (range: 1.9–65.1 months). Among 175 (52.9%) patients initially managed conservatively, 77.3% (133/172) regressed, 13.4% (23/172) persisted, 9.3% (16/172) progressed to CIN3+, and 97 (56.4%) patients achieved virological regression. 156 (47.1%) patients underwent initial excision/ablation, with an 89.4% (110/123) virological cure rate. After discharge, 7 (4.0%) and 3 (1.9%) patients redeveloped CIN in the conservative and treatment groups, respectively, during a median period of 17.2 months.

**Conclusion:**

Conservative management is a reasonable and effective management strategy in appropriately selected women with CIN2. High rates of histological and virological regression should be expected. The previously mentioned data provide useful information for deciding management options.

## 1. Introduction

Cervical intraepithelial neoplasia (CIN) is a human papillomavirus (HPV) induced precursor to cervical cancer [1]. In the UK, most CIN patients harbor high-risk HPV (HR-HPV) types 16 and 18 [2]. Up to 80% of CIN2 cases are infected with high-risk HPV, with types 16, 31, and 52 being most frequently encountered [2]. Most HR-HPV infections do not cause high-grade cervical disease and >90% resolve within two years [1, 3]. This is due to a successful immune response against HPV [3]. However, persistent HPV infections can lead to cervical cancer [4]. This is mediated through unchecked activity of HR-HPV E6 and E7 oncoproteins, allowing the accumulation of mutations leading to carcinogenesis [4].

CIN2's natural history is the least described of the three grades of CIN (CIN1, CIN2, and CIN3) [5]. It may regress to CIN1 or resolve completely, when there is a successful immune response directed toward the HPV proteins, particularly E2 and E6 [3, 5, 6]. However, failed HPV eradication leads to persistent CIN2 or CIN3+. Current UK guideline recommends excisional treatment for CIN2 and CIN3 [7]. Large loop excision of the transformation zone (LLETZ) is the commonest method [7, 8]. After treatment, test of cure (TOC) is carried out [7, 9]. Negative cytology and HPV results on TOC are reassuring and confer <1% risk of CIN2 development in the next five years [10]. However, excisional treatment is linked to increased future risks of adverse obstetric and gynaecologic outcomes, for example, cervical insufficiency, preterm delivery, and cervical stenosis [8].

Conservative management is increasingly favored for CIN2 [11]. It consists of cytology and colposcopy every 4–6 months [12]. As it spares the women excisional treatment, it is particularly attractive among young, nulliparous patients [12]. However, data on the efficacy of conservative management, when assessed by virological as well as cytological/histological parameters, have not previously been published. This study aims to investigate the outcomes of conservative management of CIN2 patients at a teaching hospital (University College London Hospital; UCLH) colposcopy unit, and where possible, compare these with the efficacy of excisional/ablative treatment. Crucially, the study incorporates HPV testing in addition to routine cytological, and where indicated, histological follow-up.

## 2. Material and Methods

This is a retrospective study using prospectively collected data on women with biopsy-proven CIN2. We included all patients referred by the national cervical screening program (NHSCSP) or their general practitioners or via internal referral within UCLH. All included patients received their first CIN2 diagnoses between 01/07/2014–31/12/2017. Exclusion criteria were as follows:

1. Biopsy reports unable to confirm definite CIN2
2. Biopsies before 01/07/2014 more severe than CIN2 (CIN3+)
3. Receiving first CIN2 diagnosis prior to the recruitment period
4. Receiving first CIN2 diagnosis at treatment (either “see and treat” or planned following prolonged low-grade cytology/biopsy/CIN1)
5. Lost to follow-up after first colposcopy appointment at which CIN2 was diagnosed on biopsy
6. Patients whose histopathology slides from elsewhere were sent to UCLH for review only and patient was managed at referring hospital

Two UCLH databases, Clinical Data Repository (UCLH in-house), and CompuScope (Iris-soft, Sale, UK) were utilized to collect data: patient demographics, cervical cytology, colposcopic impression, biopsy, smear, and HPV tests. For patients treated with excision, procedural details, excisional margins, and TOC were collected. Follow-up continued until 12 July 2019. UCLH had access then to standalone HPV testing cervical swabs (Hybrid Capture, Digene Corporation, Gaithersburg, MD), in addition to standard liquid-based cytology (LBC) HPV testing for TOC (Abbott RealTime High Risk HPV Assay, Abbott Laboratories, Abbott Park, Illinois, USA) as part of the NHSCSP.

Patients were chosen to undergo conservative management or excision/ablation at the discretion of the attending consultant. The initial conservative management group was routinely followed up every six months and underwent cervical LBC sampling, standalone HR-HPV testing, and colposcopy with or without biopsy. Follow-up visits were categorised as follows:

(1) Regression (cytological/histological): normal histology, or CIN1-only on cervical biopsy; and/or negative cytology or mild dyskaryosis or borderline nuclear changes on LBC
  - Virological regression = cytological regression and negative HPV testing
(2) Persistence: CIN2 on cervical biopsy and/or moderate dyskaryosis on LBC.
(3) Progression: CIN3 or invasive cervical cancer on subsequent cervical biopsy and/or severe dyskaryosis or suspected invasion on LBC. Progression was not based on cervical colposcopic impression.

If the patient's lesion had apparently initially improved but then reverted to high-grade CIN whilst still under colposcopy follow-up, the worst diagnosis was used for the outcome. Patients who regressed to normal histology continued conservative management until discharge. Patients on conservative management were typically followed up every 4–6 months, and discharge was decided by the attending consultant upon satisfactory clinical examination and evidence of disease regression. Follow-up was occasionally under four months at the clinician's discretion, and if patients were seen sooner with evidence of regression, they were deemed suitable for discharge at that point. Patients with cytological/histological evidence of persistent CIN2 after 4–6 months either continued conservative management or underwent excision, based on clinician's discretion and/or patient request. Patients progressing to CIN3+ were recommended to undergo excision.

The initial excision/ablation group were those advised to undergo the procedure following initial colposcopy appointment. Whilst LLETZ was the commonest treatment modality, diathermy ablation and cold knife conization (CKC) were also available according to individual patient circumstances. After treatment, patients were invited for TOC at six months, consisting of a repeat cytology and HPV test (performed on the LBC sample). In accordance with national guidance, patients were discharged if their TOC was negative for HR-HPV [7], or invited for additional follow-up if their TOC result was HPV-positive, or if their cytology was high-grade.

All standard haematoxylin and eosin histopathology slides were assessed by UCLH's team of specialist gynaecological pathologists. The slides were assessed for histological diagnosis, and depth of excision and excisional margins (involved or not involved), where appropriate. Overall margin status was “not involved” if all reported margins were not involved; otherwise, the overall status was “involved.” P16 immunohistochemical staining was not routinely used.

Patients were allocated outcomes as above according to their status at the end of colposcopy follow-up. For those still under follow-up and for outcomes subsequent to discharge, data collection was concluded on 12 July 2019. Successful conservative management (i.e., “cure”) was defined as normal histology on biopsy and/or negative or borderline/mild cytology on at least one subsequent visit, with no evidence of subsequent high-grade disease whilst still in colposcopy follow-up. Successful excisional/ablative treatment was defined as negative/borderline/mild cytology and HPV-negative on their TOC, or on at least one subsequent visit should their initial TOC fail.

Finally, the NHS OpenExeter cervical cytology database was used to collect data on smears performed outside colposcopy for both routine recall and noncompliant patients. Any records of subsequent abnormal cytology were recorded and classified as indicating a new episode of CIN. Patients not attending any of their appointments and/or who did not have any further smear reports on OpenExeter before or after their expected next smear date were classified as “lost to follow-up.”

The UK Health Research Authority Application system confirmed that formal ethical review of this audit of routine clinical practice in our institution was not required.

Descriptive statistics were used for both categorical and continuous variables where appropriate. Analytic statistics were also performed; continuous variables were compared using the independent samples Student's *t*-test, Mann-Whitney *U* test, or Kruskal-Wallis test, as appropriate. Categorical variables were analysed by Fisher's exact test or the chi-squared test, depending on minimum samples sizes requirements. Because patients were followed up for different lengths of time following diagnosis, a Cox proportional hazard regression analysis was performed, and survival curves were produced. A significance level of 0.01 was used throughout to account for multiple testing.

Data was entered into Microsoft® Excel for Mac v16.16.3 (Microsoft®, Redmond, USA) and analysed using Statistical Product and Service Solutions SPSS for Mac v.23 (IBM Corp, Armonk, New York, USA) and Stata (StataCorp. 2019. *Stata Statistical Software: Release 16*. College Station, TX: StataCorp LLC.)

## 3. Results

**Figure 1:**
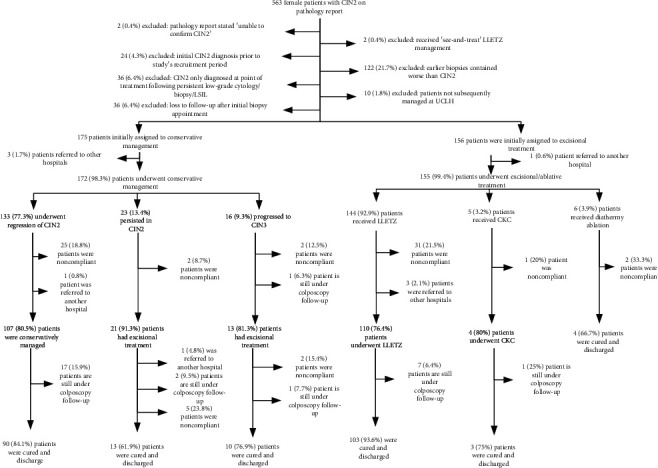
Patient recruitment chart.

The patient flow diagram (Figure 1) describes the patient recruitment. Initially, 563 patients were identified from pathology reports. 232 were excluded for the reasons shown. 331 patients were eligible for inclusion, of which 175 (52.9%) were in the initial conservative management group and 156 (47.1%) in the initial planned excisional/ablative treatment group.

The overall median follow-up was 22.6 months (range 1.9–65.1 months).  Table 1 compares the conservative and initial treatment groups. Follow-up time did not differ between the two groups (*p*=0.651). Patients initially managed conservatively were significantly younger than patients in the planned treatment group (median 27 and 28 yr, *p*=0.032) and had significantly few high-grade referral smears than patients in the planned treatment group (43/174 (24.7%) and 88/155 (56.8%), *p* < 0.001).

175 patients were initially managed conservatively; 3 were transferred to other hospitals (Table 2). Of the remaining 172 patients, 133 (77.3%) underwent cytological/histological regression and 97 (56.4%) achieved virological regression. 23 (13.4%) patients persisted as CIN2, and 16 (9.3%) progressed to CIN3. There was no statistically significant age difference among patients regressing, persisting, and progressing (*p*=0.135).

The median regression and progression times were 6.1 months (range 2.4–30.4 months) and 7.6 months (range 3.8–43.3 months), respectively. Cox regression analysis indicated that the regression time was neither influenced by smoking status (HR 1.61 (95% CI 1.06–2.44), *p*=0.025) nor parity (HR 0.86 (95% CI 0.51–1.43), *p*=0.557).

There was no evidence that progression time was influenced by smoking (HR 3.87, 95% CI (0.95–15.88), *p*=0.060) or parity (HR 1.93, 95% CI (0.53–7.09), *p*=0.321). The median interval between referral and discharge was 16.4 months (range 3.9–51.3 months), and the interval was neither significantly influenced by smoking (HR 1.31 (95% CI 0.13–12.83), *p*=0.818) nor parity (HR 2.07 (95% CI 0.21–20.4), *p*=0.534) as assessed by Cox regression. Among patients initially managed conservatively, 36 (20.9%) subsequently underwent LLETZ; 11 (30.6%) of these had CIN3 in their specimen. After treatment, all patients were invited for TOC. 32 (88.9%) patients attended and 20 (62.5%) of these had a negative result. Following further follow-up, 23 (71.9%) patients had received a negative TOC at censoring. The median time between treatment and negative TOC was six months (range 3–38 months).

156 patients were in the initial planned excisional/ablative treatment group (Table 3). 144 (92.3%) patients underwent LLETZ, 5 (3.2%) patients underwent CKC, 6 (3.8%) patients underwent diathermy ablation, and 1 (0.7%) patient was transferred to another hospital. Of patients undergoing excision (LLETZ/CKC), 93 (64.6%) had uninvolved margins. Six months after treatment, the cure rate was 73.2%. Following further follow-up (median 21.3 months, range 2.9–65.1 months) and two patients undergoing repeat LLETZ, the cure rate for all patients undergoing planned treatment (LLETZ, CKC and diathermy ablation) was 89.4% at censoring. The cure rate was significantly higher in patients aged ≤30 years old (*p* < 0.001) and in nonsmokers (*p*=0.003). There was no significant influence on the cure rate by patient age (HR 0.82 (95% CI 0.50–1.34), *p*=0.421), smoking (HR 0.80 (95% CI 0.50–1.29), *p*=0.358), parity (HR 0.89 (95% CI 0.49–1.59), *p*=0.684), endocervical margin (HR 0.63 (95% CI 0.14–2.84), *p*=0.543), ectocervical margin (HR 2.49 (95% CI 0.66–9.39), *p*=0.177), radial margin (HR 0.87 (95% CI 0.43–1.73), *p*=0.685), or overall margin status (HR 0.56 (95% CI (0.15–2.06), *p*=0.381).

Of the initial planned treatment group, the median interval between referral to discharge was 11.7 months (range: 2.9–74.8 months) and did not differ significantly from that in the conservatively managed group (median 16.4, range 3.9–51.3 months) (*p*=0.136), nor did the number of visits required prior to discharge (median 3 (range: 2–8) in the immediate treatment group and median 3 (range: 2–8) in the conservative group, *p*=0.179).

114 (60%) of women due for their next smear following discharge within the study follow-up period had a smear report on Open Exeter, the UK's national cervical cytology database. 10 (3%) patients had evidence of subsequent disease; 7 (70%) from the conservative management group; 3 (30%) from the excisional group (*p*=0.185). Overall, the median interval to subsequent dyskaryosis was 17.2 months (range: 0.2–43.9 months). The time to development of subsequent dyskaryosis did not differ significantly (median 16.9 and 23.4 months in the conservative and treatment groups, respectively, *p*=0.267). Among conservatively managed patients, smoking (HR 1.31 (95% CI 0.133–12.83, *p*=0.818) and parity (HR 2.07 (95% CI 0.21–20.40), *p*=0.534) did not significantly influence disease recurrence. To date, no patients in the conservative management group have developed cervical cancer. Among patients undergoing initial excision, none of the following factors (age at referral (HR 0.70 (95% CI 0.02–22.84), *p*=0.840), smoking (HR 0.35 (95% CI 0.02–6.90), *p*=0.490), parity (HR 3.88 (95% CI 0.22–68.33), *p*=0.354), and overall margin status (HR -, *p* ≥ 0.999) significantly predicted disease recurrence.

## 4. Discussion

Out of 172 patients managed conservatively, 133 (77.3%) patients underwent cytological/histological regression, but the histological and virological regression rates were not significantly higher among patients ≤30 years old (*p*=0.84 and *p*=0.86, respectively). This differs from a previous meta-analysis, in which regression increased to 50% over 24 months and was higher among women <30 years old [5]. However, there is little consistency in the definitions of regression, persistence, and progression in the papers cited. Our 9.3% progression rate at 60 months was lower than that in the meta-analysis, which increased from 14% at 12 months to 24% at 36 months [5].

Unsurprisingly, the virological regression rate (56.4%) was lower than the cytological/histological regression rate (77.3%). This likely reflects persistent HPV infection despite apparent CIN resolution. Such differences should be considered when designing future studies of conservative management. We argue that virological regression, rather than cytological/histological regression, should be the primary treatment objective as it defines the true conclusion of the patient's clinical disease.

Following planned initial treatment, younger age (≤30 years old) and nonsmoking status significantly increased our cure rates (*p* < 0.001 and *p*=0.003, respectively). The link between age and cure rate has been previously reported, despite unclear underlying mechanisms [13]. It is suspected that altered immunity or a positive selection towards viruses of higher oncogenic capabilities may be the cause. The link between nonsmoking and cure is also reported with smokers having thrice greater odds of treatment failure than nonsmokers [14].

We observed a higher disease recurrence risk in conservatively managed patients than in those initially undergoing treatment (70% and 30%, *p*=0.185). According to Wilkinson et al. despite having spontaneously regressed, conservatively managed patients remain at increased risk for high-grade disease for at least five years [9].

The planned treatment group presented with evidence of more high-grade smears compared with the conservatively-managed group [15]. Furthermore, initially treated patients had significantly more high-grade colposcopic impressions (*p* < 0.001) and more “CIN2 alone” on biopsy (*p* < 0.001) compared with the conservatively managed group. These histological findings have been previously reported [12].

Patients in the planned treatment group were not significantly older than conservatively managed patients, but there was a trend. This may have been a reflection of them being more likely to have true high-grade disease (as evidence by more “CIN2 only” rather than “CIN1-2” biopsies), and/or being more likely to be parous.

Our regression rate was 77.3%, while another London hospital's cohort was 57% [16]. This difference might be explained by the different regression criteria in the latter study (negative cytology on 2 consecutive visits and no high-grade colposcopic impression) [16].

We compared our results to all available similar studies from the last 10 years. A number of issues arose; some focused exclusively on patients <25 years old [6, 9, 12, 17–19], and some were laxer in their CIN2 inclusion policy, that is, inclusion of CIN2-3 as CIN2 [15, 20]. Whilst many had adopted a similar regression definition to our own, “conservative management success” was less consistently defined [9, 15]. Some were strict on requiring a certain number of consecutive negative cytology results, but others lacked such a definition [9, 15, 20, 21]. Studies varied on defining “persistence” and “progression” [6, 12, 17, 18]. Some included “CIN2-3” as persistent, rather than progressive disease [6, 12, 17, 18]. Some defined progression exclusively as invasive cervical cancer [6, 18].

With no uniform definitions, we could not compare our results directly with other studies. However, most had adopted broadly similar cytological regression definition and reported high regression rates. This, along with our results, suggests that conservative management is justified in appropriately selected CIN2 patients. This has recently been suggested by a Danish study involving >12,000 CIN2 patients managed conservatively [22].

Our posttreatment TOC cure rates were high and positively influenced by the patients' young age. This might reflect better HPV clearance in younger women and/or lower disease severity compared with older women [5].

Patients managed conservatively developed subsequent disease sooner. This was not statistically significant, possibly due to the low number of patients with sequent disease (7 and 3 in the planned conservative and treatment groups, respectively), which in itself was reassuring.

A previous meta-analysis found that involved margins increased the chances of residual and/or recurrent CIN [23]. We did not observe this in our cohort, possibly because of low numbers with recurrent disease and/or our relatively short follow-up.

This study took place within the highly-regulated UK national cervical screening program, but with the additional benefit of access to standalone HPV testing enabling us to evaluate virological regression in addition to cytological/histological regression. To our knowledge, this is the UK's largest reported CIN2 cohort, one of the larger studies reported worldwide and crucially, the first to report on virological regression rates. Finally, our study adds to the limited published data comparing the outcomes of conservative and excisional/ablative treatments for CIN2 in the same cohort, of which ours is the only such study to have investigated virological regression [6, 9, 12, 15–18, 21, 24].

The study has several limitations. 21.4% of included patients (70/331) were noncompliant with and/or lost to follow-up, and this is in combination with our median follow-up of 22.6 months, meaning that we may have underestimated the true disease recurrence rate. There was incomplete information on some risk factors, such as smoking status. Despite this, smoking was still found to be significantly inversely associated with successful TOC. The clinical HPV assays used for testing differed in the posttreatment tests done as part of the national program and those done as standalone tests in the conservatively managed group, although this was unlikely to introduce significant bias, given the equivalence of the tests used [25]. The study's retrospective nature made it hard to pinpoint the exact reason why some patients were offered conservative management rather than initial treatment. Thus, it was a clinician's clinical decision as to whether their assessment made it reasonable to offer conservative management (in collaboration with the patient). This clearly would have introduced selection bias, as evidenced by the higher proportion of high-grade colposcopic appearances and “CIN2-only” biopsies in the initially treated group compared with the conservatively managed group. In addition, planned follow-up schedules of the conservatively managed group, and decisions to undergo subsequent treatment were down to clinician/patient preferences, rather than a clinical trial protocol. Also, this audit of the outcomes of routine clinical protocol at our hospital may not be generalizable to other healthcare settings. Finally, the absence of routine p16 immunostaining in equivocal cases of CIN2 means we cannot be certain that all the “CIN2” cases were high-grade according to the Lower Anogenital Squamous Terminology (LAST) criteria [26].

To conclude, conservative management consisting of regular colposcopy follow-up, combined with biopsy, cervical smear, and HPV testing is a reasonable and effective management strategy for appropriately selected patients with CIN2, preferably nonsmoking with low-grade or normal colposcopy impression and referred with low-grade or normal cytology. High rates of histological and, in particular, virological regression of CIN2 should be expected. Furthermore, our data provide useful information for clinicians and patients when deciding management options in the era of HPV testing [27].

## Figures and Tables

**Table 1:** Demographics, risk factors, and clinical results from colposcopy.

Variables	Initial conservative management (*n*: 172)	Initial excisional treatment (*n*: 155)	*p* value
Age at Referral (median; range)	27; 21–43	28; 22–65	0.032
Smoking			0.362
Yes	50 (28.6%)	56 (35.9%)	
No	114 (65.1%)	91 (58.3%)	
Not available	11 (6.3%)	9 (5.8%)	
Referral LBC results			
Borderline changes (squamous/endocervical)	20 (11.4%)	14 (9.0%)	<0.001
Mild dyskariosis	94 (53.7%)	41 (26.3%)	
Moderate dyskariosis	34 (19.4%)	44 (28.2%)	
Severe dyskariosis	9 (5.1%)	44 (28.2%)	
Clinical referrals (cytology not applicable)	17 (9.7%)	12 (7.7%)	
Not available	1 (0.6%)	1 (0.6%)	
Biopsy			
Transformation zone			0.065
1	122 (69.7%)	89 (57.1%)	
2	23 (13.1%)	29 (18.6%)	
3	1 (0.6%)	4 (2.6%)	
Not available	29 (16.6%)	34 (21.8%)	
Colposcopy impression			<0.001
High-grade	37 (21.1%)	89 (57.1%)	
Low-grade	124 (70.9%)	57 (36.5%)	
Normal	3 (1.7%)	4 (2.6%)	
Others	9 (5.1%)	6 (3.8%)	
Not available	2 (1.1%)	0	
Biopsy result			<0.001
CIN1-2	102 (58.3%)	41 (26.3%)	
CIN2	73 (41.7%)	115 (73.7%)	
LBC at biopsy (if done)			0.005
Borderline changes (squamous/endocervical)	7 (19.4%)	2 (5.1%)	
Mild dyskariosis	9 (25.00%)	10 (25.6%)	
Moderate dyskariosis	6 (16.7%)	11 (28.2%)	
Severe dyskariosis	0	10 (25.6%)	
Negative/Normal	14 (38.9%)	6 (15.4%)	
HPV test at biopsy (if done)			0.022
Positive	44 (73.3%)	49 (94.2%)	
Negative	16 (26.7%)	3 (5.8%)	
Median follow-up (range)	23.4 months (1.9–55.9)	21.3 months (2.9–65.1)	0.982

**Table 2:** Risk factors and other characteristics in initial conservative management group.

Variables	Total	Regression	Persistence	Progression	*p* value across 3 groups
Number of patients	172	133 (77.3%)	23 (13.4%)	16 (9.3%)	
Median age at referral (years) (range)	27.5 (21–46)	28 (21–46)	28 (24–42)	25.5 (24–37)	0.135
Smoking					N/A
Yes	50 (29.1%)	38 (28.6%)	7 (30.4%)	5 (31.3%)	
No	111 (64.5%)	85 (63.9%)	15 (65.2%)	11 (68.8%)	
Not available	11 (6.4%)	10 (7.5%)	1 (4.3%)	0 (0%)	
Parity					N/A
Nulliparous	147 (85.4%)	116 (87.2%)	19 (82.6%)	12 (75.0%)	
Parous	25 (14.5%)	17 (12.8%)	4 (17.4%)	4 (25.0%)	
Immunosuppression					N/A
Yes	4 (2.3%)	4 (3.0%)	0	0	
No	168 (97.7%)	129 (97.0%)	23 (100%)	16 (100%)	
Median Interval between referral and discharge (months) (range)	16.4 (3.9–51.3)	17.2 (3.9–43.2)	15.1 (4.5–51.3)	18.1 (13.3–36.8)	0.529
Median number of visits prior to discharge (range)	3 (2–8)	3 (2–6)	4 (4–8)	4 (4–6)	<0.001

N/A = not applicable.

**Table 3:** Summary of characteristics of initial excision patients.

Variables	Total	LLETZ	Cold knife cone (CKC)	Diathermy ablation	*p* value across 3 treatment groups
Number of patients	155	144 (92.9%)	5 (3.2%)	6 (3.9%)	
Median age at referral in years (range)	28 (22–65)	28 (22–63)	42 (29–65)	24 (22–29)	0.003
Smoking					
Yes	56 (100%)	51 (91.1%)	2 (3.6%)	3 (5.3%)	0.502
No	90 (100%)	85 (94.4%)	3 (3.3%)	2 (2.2%)	
Not available	9 (100%)	8 (88.9%)	0	1 (11.1%)	
Excisional margins					
Endocervical margin					0.234
Not involved	132 (88.6%)	129 (89.6%)	3 (60%)	N/A	
Involved	9 (6.0%)	8 (5.6%)	1 (20%)	N/A	
Not available	8 (5.4%)	7 (4.8%)	1 (20%)		
Ectocervical margin					>0.999
Not involved	102 (68.5%)	99 (68.8%)	3 (60%)	N/A	
Involved	38 (25.5%)	37 (25.7%)	1 (20%)	N/A	
Not available	9 (6.0%)	8 (5.5%)	1 (20%)		
Radial Margin					>0.999
Not involved	117 (78.5%)	113 (78.5%)	4 (80%)	N/A	
Involved	9 (6.0%)	9 (6.3%)	0	N/A	
Not available	23 (15.4%)	22 (15.3%)	1 (20%)		
Overall Margin					0.615
Not involved	93 (64.6%)	91 (65%)	2 (50%)	N/A	
Involved	51 (45.4%)	49 (35%)	2 (50%)	N/A	
TOC					0.049
Yes	123 (79.4%)	116 (80.6%)	3 (60%)	4 (66.7%)	
No	32 (20.6%)	28 (19.4%)	2 (40%)	2 (33.3%)	
Cure rates 6 months posttreatment	73.2% (90/123)	72.4% (84/116)	66.7% (2/3)	100% (4/4)	
Overall cure rates (as of July 2019)	89.4% (110/123)	88.8% (103/116)	100% (3/3)	100% (4/4)	0.805
Median interval between referral and discharge (months) (range)	11.7 (2.9–74.8)	11.4 (2.9–74.8)	15.7 (8.8–40.2)	16.8 (13.5–29.6)	0.286
Median number of visits prior to discharge (range)	3 (2–8)	3 (2–8)	6 (3–8)	3 (3–4)	0.021

## Data Availability

A summary of anonymized data is available upon reasonable written request to the authors.
